# Opioid use disorder and treatment: challenges and opportunities

**DOI:** 10.1186/s12913-019-4751-4

**Published:** 2019-11-25

**Authors:** Kim A. Hoffman, Javier Ponce Terashima, Dennis McCarty

**Affiliations:** 1 Oregon Health and Science University-Portland State UniversitySchool of Public Health, 3181 SW Sam Jackson Park Rd., CB669, Portland, OR 97239-3088 USA; 2 Case Western University-University Hospitals, Cleveland, OH USA

## Abstract

**Background:**

Addiction health service researchers have focused efforts on opioid use disorder (OUD) and strategies to address the emerging public health threats associated with the epidemics of opioid use and opioid overdose. The increase in OUD is associated with widespread access to prescription opioid analgesics, enhanced purity of heroin, the introduction of potent illicit fentanyl compounds, and a rising tide of opioid overdose fatalities. These deaths have become the face of the opioid epidemic.

**Main Text:**

OUD is a chronic disorder that usually requires both medications for opioid use disorder (MOUD) and psychosocial treatment and support. Research has found that MOUD with an opioid receptor agonist (methadone), partial agonist (buprenorphine), or opioid antagonist (extended-release naltrexone) can support recovery. Despite compelling evidence that MOUD are effective, they remain underutilized. More research is needed on these therapies to understand the feasibility of implementation in clinic settings.

**Conclusion:**

This special issue focuses on how health services research has emerged as an important contributor to efforts to control the opioid epidemic in North America and Europe.

## Background

*BMC Health Services Research* is pleased to release a special issue focused on health services research addressing opioid use and opioid use disorders. Addiction health services research, an emerging specialization within the broad area of health services research, examines how social factors, financing systems, organizational structures and processes, health technologies, and personal beliefs and behaviors affect access to and utilization of health care, the quality and cost of healthcare, and in the end, our health and well-being. Multidisciplinary addiction health services research draws on tools from epidemiology, biostatistics and public health, theories from social science (e.g., geography, psychology, sociology), medical expertise (e.g., anesthesiology, cardiac surgery, emergency medicine, family medicine, internal medicine, psychiatry), legal and policy expertise, and economic analyses to assess the quality of care and impacts on quality of life. Ultimately, the goals of addiction health services research are to identify the most effective ways to organize, manage, finance and deliver high quality care [1, 2]. In the papers in this special issue, health service researchers report on the opioid epidemic and strategies to address and control it. For example, overdose rescue using naloxone distribution has become an effective community intervention; Papp summarizes the use of naloxone rescue kits in Ohio, a state that has reeled from the impacts of the opioid overdoses. Within community-based opioid treatment programs, Becker assessed user-centered design principles to gather qualitative data on familiarity with contingency management, current clinical practice, and preferences regarding the implementation of contingency management. Priest discusses strategies to initiate opioid agonist therapy among hospitalized patients. Hoffman provides a qualitative assessment of the introduction of extended-release naltrexone in HIV primary care for patients with opioid use disorders and the challenges of using an opioid antagonist therapy. Although we know that medications for opioid use disorder (MOUD) are effective in treating opioid use disorders (OUD), retention in treatment can be difficult; Beamish et al. describe the planning and implementation of a quality improvement initiative aimed at keeping people in care through changes to workflow and care processes in Vancouver, Canada.

Opioids are natural or synthetic chemicals that bind to opioid receptors in the central nervous system and can reduce feelings of pain. The 1961 Single Convention on Narcotic Drugs (United Nations) classifies opioids as narcotics and regulates medical use, distribution and access through the International Narcotics Control Board (INCB). Opioids have the potential for misuse and addiction. Worldwide, use of opioid analgesics doubled between 2001 and 2003 and 2011–2013 [3]. There is growing concern about the misuse of opioids in Africa, in particular the access and use of Tramadol [4] but the public health emergency is most apparent in North America and parts of Europe [5]. This increase in misuse is associated with widespread access to prescription opioid analgesics, enhanced purity of heroin, the introduction of potent illicit fentanyl compounds, and a rising tide of opioid overdose fatalities.

In the United States, which leads the world in opioid consumption, prescriptions for hydrocodone and oxycodone greatly increased in the late 1990s [6]. Though mostly used for cancer related pain, opioids are also commonly prescribed as a treatment for chronic and acute non-cancer pain conditions [7] despite controversies about their effectiveness and safety with long-term use [8], adverse effects [9, 10], loss of analgesic effectiveness of the drug with long term use [11], addiction potential [12, 13], and drug diversion [14]. Prescription drug abuse is the fastest growing drug problem in the U.S; up to 1 in 4 people receiving long-term opioid therapy in a primary care setting can struggle with a moderate to severe opioid use disorder [15–17]. According to the Centers for Disease Control, important factors responsible for the OUD epidemic include patients receiving more than one prescription from multiple providers or taking very high doses of the medication [18]. These trends were seen in other developed countries; for example, between 1992 and 2012, opioid dispensing episodes increased 15-fold in Australia [19] and total prescription opioid analgesic dispensing increased across Canada until 2011 [20].

More than 700,000 individuals died from a drug overdose between 1999 and 2017 in the U.S. [18]. Drug overdose fatalities exceeded 70,000 in 2017 and two-thirds (68%) was attributable to opioids [21]. Synthetic opioids were involved in almost 60% of all opioid-involved overdose deaths; a 45% increase from 2016 to 2017 [21]. Deaths from heroin related overdoses remained relatively stable in 2017 at just over 15,000 deaths [21]. There are increasing concerns about the involvement of synthetic opioids in drug overdoses, in particular, illicit fentanyl sold in the heroin market [22]. Most deaths due to fentanyl originate from illicitly produced fentanyl, not prescribed fentanyl [23].

In 2013, the U.S. Food and Drug Administration raised concerns about the risks of overprescribing long-acting opioid analgesics. Long acting opioid formulations may contain three times the dose of immediate release tablets and can lead to respiratory depression and death when crushed, injected or taken with alcohol [24]. Controversy exists about the risk of abuse with short and long acting formulations. The controlled-release oral formulations are aimed to reduce the abuse liability due to a gradual onset and sustained delivery of medication; however, abuse may develop with these formulations because users may misuse or tamper with the formulation to evade the gradual release feature. Furthermore, sustained release oxycodone product has a bi-phasic release pattern such that its initial speed of delivery begins to approach that of immediate-release oxycodone [25]. Thus, patients may mistakenly believe that extended-release products are safer [26]. A greater risk of abuse and overdose is seen among patients who receive multiple opioid prescriptions, overlapping opioids, overlapping opioids and benzodiazepines and opioids at high dosage levels [27, 28]. Unsafe and high risk prescriptions of opioids may be linked to deficiencies in the management of pain conditions in different populations, including those at risk of addiction, and difficulties adapting guidelines to patients who have multiple pain disorders [29].

The opioid crisis provides opportunity to develop and test new theories to ameliorate the harms of drug use and to invent tools that are applicable to the emergence of new drugs (e.g., methamphetamine and synthetic cannabinoids) and new drug use challenges (e.g., vaping). Addiction health services research tracks and assesses opioid use and misuse around the globe. The United Nations Office on Drugs and Crime updates its *World Drug Report* annually tracking use of amphetamine type stimulants, cannabis, cocaine, opioids and other drug use and problems associated with the production, manufacturing and use of illicit substances [30]. Similarly, the European Monitoring Centre for Drugs and Drug Addiction tracks overdoses, mortality and other problems associated with drug and opioid use. Health service investigators are reporting on levels of opioid misuse in Australia [19, 31], Brazil [32] and Southeast Asia [33, 34], which are also are seeing troubling levels of opioid misuse.

### Treatment for opioid use disorder

Opioid use disorder is a chronic disorder that often requires both medication for opioid use disorder (MOUD) and psychosocial treatment and support. Rigorous research has found that MOUDs with an opioid receptor agonist (methadone), partial agonist (buprenorphine), or opioid antagonist (extended-release naltrexone) can facilitate recovery from opioid use disorders [35]. Methadone has been widely used since the 1960s. Buprenorphine, a partial opioid agonist with a better safety profile, was introduced in France in the 1990s and approved in the US in 2002. MOUDs work by reducing withdrawal symptoms and opioid cravings while decreasing the biological response to future drug use. Individuals receiving MOUDs cease or decrease their use of injection drugs and thus lower their rates of contracting infectious diseases. A recent report by the National Academies of Science, Engineering and Medicine found that individuals undergoing long term treatment with methadone or buprenorphine reduced the risk of death 50% [36]. Two clinical trials found that extended release naltrexone and buprenorphine inhibited return to use when patients initiated medication in inpatient or residential detoxification programs [37, 38].

MOUD treatment is often coupled with counseling and behavioral therapies, such as Cognitive Behavioral Therapy. In the U.S., federal regulations require centers that dispense methadone to provide counseling and federal legislation encourages physicians who prescribe buprenorphine to refer patients for counseling [39]. Despite these requirements, there are no counseling approaches specifically designed for patients with opioid use disorder and therapists are frequently not using evidence-based psychosocial interventions. Participation in individual and group therapy, moreover, can help patients remain engaged in their recovery and inhibit return to use. Although some patients have successfully maintained abstinence using only psychosocial approaches, counseling without support from MOUD is often associated with a return to use [40].

Despite compelling evidence that MOUD is effective, these medications remain underutilized. This is due in part to the need for daily dosing for most of the medications. Recent advances are changing the landscape, however. Extended-release naltrexone (XR-NTX), a deep muscle injection that lasts 28 days, eliminates the need for daily dosing. A once-monthly buprenorphine injection, Sublocade®, became FDA approved in 2017 and Probuphine®, an implantable buprenorphine product, was approved in 2016. Both medications can improve treatment retention. However, limited access to these and other MOUDs has hindered efforts to address the opioid addiction epidemic [41]. Only 36% of specialty substance use disorder treatment organizations in the U.S. provided any of the FDA approved MOUDs [42]. Additionally, more research is needed on these therapies to understand the feasibility of implementation in primary care and correctional settings.

### Main body

Addiction health services research has emerged as a contributor to efforts to control the opioid epidemic in North America and Europe. Health surveillance systems monitor trends in opioid overdose and the shift in the epidemic from illicit heroin to prescription analgesics to illicitly manufactured fentanyl and its analogues. A Comparison of Canadian and U.S. policies regulating use of opioid agonist therapies suggested that the limits on methadone and buprenorphine in the U.S. are antiquated and that individuals with opioid use disorders can benefit from additional opioid agonist therapies [43]. European [44] and Canadian [45] trials of diacetylmorphine document the value of using pharmaceutical heroin as an opioid agonist therapy for individuals who are non-responsive to methadone or buprenorphine. In the U.S., the Affordable Care Act and Medicaid expansion facilitated access to treatment for opioid use disorders [46–49].

Policies and guidelines are increasingly proposed and adopted to address the opioid epidemic. Given the mounting burden on the public due to the misuse of opioids, public health institutes such as the U.S. Centers for Disease Control and Prevention promote the adoption of standards regarding the prescription of opioids. Their prescriber guidelines address three facets: 1) determining when to initiate or continue opioids for chronic pain outside of active cancer treatment, palliative care, and end-of-life care; 2) opioid selection, dosage, duration, follow-up, and discontinuation; and 3) assessing risk and addressing harms of opioid use [7]. The guidelines also include helpful instructions for patients on the limitations and consequences of use including addiction and overdose. A recent commentary on the implementation of these guidelines notes that the guidelines have been an effective tool but providers must also make their individual clinical decisions based on each patient’s unique circumstances [50].

In general, there is a need to find a balance between policies that prevent opioid misuse, abuse, addiction and overdose while at the same time supporting patient needs for appropriate pain medications. Physicians prescribing opioids should advise patients about serious adverse effects of opioids, particularly the development of a potentially serious lifelong opioid use disorder [7]. Providers should be aware of how to screen for OUD and, if a disorder is detected, understand how to treat their patient or refer to a reputable treatment program. In addition to verbal education, policies related to written instruction can be enacted. For example, current labeling of opioids in the U.S. includes detailed instruction that opioids should only be used when other measures to limit pain have been unsuccessful, the risks associated with the use of opioids, the need for monitoring by an expert provider who can discuss regularly when the use of opioids can be stopped, and that the drug should only be dispensed in limited quantities [51].

Another strategy includes requiring opioid manufacturers to fund continuing medical education (CME) to providers at low/no cost. These are voluntary programs. In the U.S., the Food and Drug Administration mandates education for all prescribers, although obstacles remain to fully enacting this requirement [51]. Additionally, policy makers can review coverage for non-pharmacological pain management (e.g., cognitive behavioral therapy, physical therapy, rehabilitative exercise) and evaluate how current pain management practices and policies (especially concerning complex chronic non-cancer pain) impact patients.

Resources should be invested to ensure that opioid prescriptions are accurately recorded and monitored, so that intervening steps can be taken if problematic patterns are found. Prescription Drug Monitoring Programs can provide prescribers and pharmacies with information that can identify drug seeking, patient safety or patients at risk for opioid use disorder [52]. PDMPs are databases that track controlled substance prescriptions at a regional level (e.g., country, state, province) and can be useful as a public health tool. Health departments can follow the patterns of the epidemic and this can inform programmatic interventions. Data can also be used to generate reports that can identify inappropriate prescribing trends; responses can then be carried out to address “hot spot” areas that are contributing to the epidemic. These areas can also be targeted for systems that address overdose risk reduction interventions and distribution of naloxone, an effective drug for reversing opioid overdoses [53]. Results from overdose response programs are showing some effectiveness in preventing overdose-related deaths [54].

## Conclusions

Given developments in the field of OUD prevention and treatment, there is cause for hope in the face of this epidemic. Stable and secure funding is required for evidence-based treatment, evaluation and development of pharmacotherapies to treat drugs of abuse, and assessment of changing policies and policy impacts. Addiction health services research can continue to a) assess the effects of government policies on access to care and access to prescription analgesics, b) monitor changes in the markets for and manufacturing of illicit opioids c) evaluate systems of care for opioid use disorders to enhance treatment access and effectiveness, d) document and monitor the impacts of harm reduction interventions (e.g., syringe exchange and safer injection sites), e) assess prejudice and bias (i.e., stigma) toward people who use drugs and f) measure the economic costs associated with drug use.

## Data Availability

Not applicable

## Abbreviations

MOUD: Medication for opioid use disorder
OUD: Opioid use disorder
TAU: Treatment as usual
XR-NTX: Extended release naltrexone

## Availability of data and materials

De-identified transcripts are available from the corresponding author.
